# Factors associated with successful transition among children with disabilities in eight European countries

**DOI:** 10.1371/journal.pone.0179904

**Published:** 2017-06-21

**Authors:** John Ravenscroft, Kerri Wazny, John M. Davis

**Affiliations:** 1 The Scottish Sensory Centre, Moray House School of Education, University of Edinburgh, Edinburgh, United Kingdom; 2 Centre for Global Health Research, Usher Institute of Population Health Science and Informatics, University of Edinburgh, Edinburgh, United Kingdom; Universidad de Castilla-La Mancha, SPAIN

## Abstract

**Introduction:**

This research paper aims to assess factors reported by parents associated with the successful transition of children with complex additional support requirements that have undergone a transition between school environments from 8 European Union member states.

**Methods:**

Quantitative data were collected from 306 parents within education systems from 8 EU member states (Bulgaria, Cyprus, Greece, Ireland, the Netherlands, Romania, Spain and the UK). The data were derived from an online questionnaire and consisted of 41 questions. Information was collected on: parental involvement in their child’s transition, child involvement in transition, child autonomy, school ethos, professionals’ involvement in transition and integrated working, such as, joint assessment, cooperation and coordination between agencies. Survey questions that were designed on a Likert-scale were included in the Principal Components Analysis (PCA), additional survey questions, along with the results from the PCA, were used to build a logistic regression model.

**Results:**

Four principal components were identified accounting for 48.86% of the variability in the data. Principal component 1 (PC1), ‘child inclusive ethos,’ contains 16.17% of the variation. Principal component 2 (PC2), which represents child autonomy and involvement, is responsible for 8.52% of the total variation. Principal component 3 (PC3) contains questions relating to parental involvement and contributed to 12.26% of the overall variation. Principal component 4 (PC4), which involves transition planning and coordination, contributed to 11.91% of the overall variation. Finally, the principal components were included in a logistic regression to evaluate the relationship between inclusion and a successful transition, as well as whether other factors that may have influenced transition. All four principal components were significantly associated with a successful transition, with PC1 being having the most effect (OR: 4.04, CI: 2.43–7.18, p<0.0001).

**Discussion:**

To support a child with complex additional support requirements through transition from special school to mainstream, governments and professionals need to ensure children with additional support requirements and their parents are at the centre of all decisions that affect them. It is important that professionals recognise the educational, psychological, social and cultural contexts of a child with additional support requirements and their families which will provide a holistic approach and remove barriers for learning.

## Introduction

The European Union (EU) and its member states have endeavoured to improve the social and economic situation of people with disabilities. The EU has signed up and implemented a series of important legislative Charters and conventions. For example, Article 1 of the Charter of Fundamental Rights of the EU (the Charter) states that ‘Human dignity is inviolable. It must be respected and protected [1].’ In addition, Article 26, states that ‘the EU recognises and respects the right of persons with disabilities to benefit from measures designed to ensure their independence, social and occupational integration and participation in the life of the community.’ This coupled with the European Disability Strategy 2010–2020 [2] and the UN Convention of the Rights of the Child (1989)[3] place the voice of children at the heart of any process that involves them.

In order to support independence and participation into the community, children with disabilities, and in particular children with complex additional support requirements need to be appropriately included into the national education system with each child provided with individual support. Part of the process to ensure the best outcomes for children with complex additional support requirements is to support children through periods of school transition which should be considered as a social process [4]. School transitions are also influenced by the different contexts and ecological systems that are experienced by the child [5]. Following a rights-based approach as outlined by the EU and indeed other organisations such as UNICEF, one would expect to see the child taking a primary and leading role in the transition process [6]. Indeed, there is now a global policy expectation that children and young people are, and should be, essential actors in finding solutions to their own life issues. However, there exists a gap in research knowledge concerning how to take a child centred approach to the differing forms of school transition (early years to primary, primary to secondary, and secondary to non-school destinations). This gap in the literature becomes even more apparent when we look at child centred collaborative approaches to transition when the child has disabilities with complex additional support needs [7]. There is a paucity in data examining what we can learn from children with complex support needs and their parents, in the transition process when exiting from existing segregated systems into proactive innovative learning environments for all learners. This learning from children with additional support needs (in Scotland this term is used rather than special needs) should enable the transition of children to go beyond simple mainstreaming but to a shared mutual learning benefit for disabled and non-disabled students alike.

There are a range of psychological works that put forward a broadly deficit model of transition that are steeped in psychological notions. [8–13]. These writings suggests for example, that transition is a natural part of child-development, involves a sense of loss, is stressful for children and is connected to wider issues such as parenting, school systems and policy environments [14–16].

The traditional approach of transitioning children with complex additional support needs has been to engage with only specific aspects of administrative and delivery modalities within systems that remain distinct and separate [17]. Elsewhere, we have identified that innovation, in the area of transition of children with complex support needs, can be blocked by professionals who overly value adult expertise. We have suggested that blockages occur because professional practice is often too focussed on these psychological ideas (e.g. age and stage) that lack a child centred philosophy or notions of complexity/fluidity. Similarly, psychological writing on transition has been critiqued for being reducing disabled children down to the role of victim, negating their ideas on change, over stressing negativity/vulnerability rather than resilience and/or assuming disabled children lack competence, to be involved in participative ways [18].

The main objective for this research sought to statistically identify and understand the possible factors that are drivers of successful transition of children with complex additional learning support requirements. This research also conducted qualitative interviews and focus groups with parents, professionals and pupils which we have reported elsewhere [19].

In order to identify the drivers for successful transition a range of research questions were devised to quantitatively test the theory of child and parent-led processes of transition. Partner countries of this research work had been influenced by the UK disability movement [20–21], where inclusion does not mean treating all children the same; rather, it involves ensuring that the structures, cultures and relationships in mainstream schools remove the barriers to learning that children with additional needs encounter so that they can enjoy equity of experience with other children [22]. Similarly, we have argued this requires disabled children to be involved in forums and process that enable collaborative problem solving with adults and that disabled children should be enabled to take leadership roles in process of change [23].

This research thus aimed to inform the development of inclusion, transition collaborative working and child led participation by trying to identify best practice among, 8 EU member states (Bulgaria, Cyprus, Greece, Ireland, the Netherlands, Romania, Spain and the UK). The consortium of the 8 European countries were constructed from partners where each partner within each country demonstrated awareness of the importance of transition and the role of teamworking in achieving inclusion for students with additional support needs. All partners had an understanding of sustainable outcomes and results that make a definable difference for participation in learning. The 8 partners differed in stages of applying various approaches from an inclusive education agenda. The Netherlands and Bulgaria developed policies on inclusion that have at their heart a presumption of mainstreaming. In Spain, the general principles of inclusive education were utilised to suggest that whenever possible and appropriate students’ should be educated in mainstream school environments. The consortium of partners had also an understanding of additional support needs. For example, in Cyprus and Greece approaches were based on ideas that centred on the pupils’ needs and teachers strategies and were underpinned by the idea that children should experience success and should be encouraged to believe in themselves. In the UK and Ireland we see the development of child centred approaches. Due to these differing yet similar approaches to the education of children with additional support needs, these group of countries provided a wide range of inclusive concepts and practices in which to test the research questions.

### Research Question 1

Is Inclusion for all connected to structures cultures and relationships that promote participatory discussion and collaborative problem solving between children, parents and staff [24–25]?

### Research Question 2

Does positive transition and inclusion for all result from children with complex additional support needs having autonomy and leading activities of transition within a context that promoted creative social relations [26]?

### Research Question 3

Is a child’s involvement in peer support and recreational activities key to positive transition and inclusion [27]?

### Research Question 4

Does planning, provision of resources, development of flexible curriculum, teacher strategies and information sharing ahead of time lead to positive transition and inclusion [28]?

### Research Question 5

Are participatory goal setting, early development of plans and regular review of plans and service delivery essential aspects of positive transition and inclusion [29–30]?

In developing these questions considerations arose whether child-led and parent-led process of transition as indicated by UNICEF and the United Nations Convention on the Rights of the Child (UNCRC) were complementary or not and this could be of particular concern in countries where there was not a tradition of involving disabled children in processes of decision making. Could we therefore attempt to make recommendation at a EU level concerning child-led and parent-led process of transition?

## Methods

### Participants

Parents of children with complex additional support needs from 8 different EU member states participated in this survey. Participants were located in Bulgaria, Cyprus, Greece, Ireland, the Netherlands, Romania, Spain and the United Kingdom.

### Procedure

Organisations in eight EU member states contacted primary and secondary schools that had children with additional support requirements in every country. The schools were individually selected by each member partner as having already transitioned children with additional support needs into and or out of their schools. Schools that did not have this experience, were not chosen to participate. Schools acted on the study’s behalf to identify parents who may wish to participate, providing them with information sheets and consent forms given to them by each partner organisation.Parents were then asked to contact the study organisers if they wished to participate and to complete the consent forms which then sat with the organisations. Parents completed the on line questionnaire anonymously to the research team. The study had ethical approval from the Moray School of Education Ethics Committee at the University of Edinburgh. The study took place during the years 2014–2016. There was no remuneration provided to participants or to the schools assisting with recruitment and Table 1 shows the organisations involved in participant recruitment.

**Table 1:** Organisations involved in participant recruitment.

Organisation	EU Member State
University of Edinburgh + City of Edinburgh Cowgate Nursery	United Kingdom
Euro Ed	Romania
The University of Nicosia	Cyprus
Panton Schools	Greece
Centrum voor Maatschepelijke Ontwikkeling (CMO) Gronigen + OPDC de Wilgenborgh	The Netherlands
I-E Pi del Burgar	Catalonia/Spain
The Centre for Inclusive Education	Bulgaria
Enable Ireland Disability Services	Ireland

### Survey

The survey was conducted online through Survey Monkey. Respondents had the option to conduct their surveys in English, Bulgarian, Romanian, Catalonian or Greek.

The survey consisted of 41 questions (40 questions used in the analysis) [S1 File] initially developed and built upon previous questionnaires developed by Davis [31–32]. The survey questions were then further refined through consultation with the organisations listed above.

The questionnaire was validated in all 8 member countries and time was spent on ensuring the questions asked were culturally sensitive and appropriate. This was achieved by initially carrying out three research team meetings with all member countries present to analyse the cross-cultural comparability of the research instrument. Once agreed all questions were then professionally translated into the appropriate language for each country. Each partner from the relevant country then checked each question for accuracy of translation. Once this process had been conducted and all countries agreed on the final set of ‘translated’ questions, a pilot of the questionnaire took place within each country.

The pilot required each partner organisation to identify three parents (3 parents X 8 partners = 24 pilot responses) to complete the questionnaire, and to report back to the organisation. Each organisation then reported back to first and third author regarding any changes. As a result of the pilot, minor changes to the wording of one question in the Netherlands took place. Two changes of wording for the Spanish version of the questionnaire and in Romania and Bulgaria an additional explanation of “therapist” took place. No questions were removed from the initial questionnaire because of the pilot responses. We were then able to employ a uniform format for questionnaires across all 8 countries.

In addition to demographic information, information was collected on: parental involvement in their child’s transition, child involvement in transition, child autonomy, school ethos, professionals’ involvement in transition and integrated working, such as, joint assessment, cooperation and coordination between agencies. Survey questions that were designed on a Likert-scale were included in the Principal Components Analysis (PCA), additional survey questions, along with the results from the PCA, were used to build a logistic regression model.

### Principal components analysis

Exploratory principal components analysis (PCA) was conducted on 306 observations for categorical survey questions, using a correlation matrix. The KMO and Barlett’s test were run on the correlation matrix. Through examination of the scree plot and use of Kaiser’s criterion, we extracted 4 components. The number of large residuals, mean number of residuals and a histogram of the residuals were examined to ensure we had extracted the correct number of components. We employed an oblique rotation, using “oblimin,” as we expected our data to be highly correlated. We used a cut-off of 0.3 to interpret the factors.

### Logistic regression

The factor scores from the principal components were used in a logistic regression along with the survey questions that were relevant to the entire population. The regression was built using a step-wise model. Model fit was examined using the Akaike Information Criterion (AIC) and the null and residual deviances. A model Chi square statistic determined that the model significantly predicted the fit better than a null model and the Hosmer-Lemeshaw test was not significant. There were no large residuals, no multicollinearity, the assumption of independence was met and the diagnostic tests for influential measures were not problematic. All analyses were completed in R Studio.

## Results

306 parents of children, ranging from 3–13 years, with additional support needs from Bulgaria, Cyprus, Greece, Ireland, the Netherlands, Romania, Spain and the United Kingdom participated in the survey [S2 File].

Descriptive characteristics of the respondents and their children can be found in Table 2.

**Table 2:** Descriptive characteristics of respondents and their children.

Characteristic Category	Characteristic (N)^1^
**Country**	Bulgaria (54)
	Cyprus(49)
	Greece(50)
	Ireland (35)
	Netherlands (2)
	Romania (29)
	Spain (35)
	United Kingdom (9)
**Language**	Bulgarian (62)
	Catalonian (35)
	English (157)
	Greek (30)
	Romanian (22)
**Parent Gender**	Female (145)
	Male (181)
**Child Gender**	Female (82)
	Male (118)
**Child Age**	3–5 (45)
	6–8 (75)
	9–11 (74)
	12–13 (69)
**Support Requirement**	Physical (12)
	Intellectual (28)
	Sensory (40)
	Learning (42)
	Developmental (21)
	Autism (31)
	Unknown (15)
	Other (10)
	Multiple (64)
**School Attending**	Mainstream Preschool (26)
	Special Class in a Mainstream Primary School (23)
	Mainstream Primary School (83)
	Special Primary School (24)
	Mainstream Secondary School (47)
	Special Secondary School (11)

^1^ (absolute numbers in parentheses)

Four principal components were identified, displayed in Table 3. Together, the components are responsible for 48.86% of the variability in the data. Despite having some overlapping questions contributing to the components, none of the components are strongly correlated with one another.

**Table 3:** Results from principal components analysis.

Question	Child Inclusive Ethos (PC1)	Parental Involvement (PC3)	Planning & Coordination (PC4)	Child Autonomy & Involvement (PC2)
**Child’s involvement in peer support**	0.76			
**Child included in recreational activities**	0.69			
**Staff works as a team**	0.67			
**School inclusiveness**	0.61			
**Curriculum flexibility**	0.56			
**Extent child adjusted**	0.47	0.47		
**Information transfer**	0.42			
**Accessibility of the school**	0.41	0.39		
**Parental involvement in school decision-making**	0.37			0.31
**Contact with school and agencies**		0.74		
**Parental involvement in transfer decision**		0.74		
**Parents’ involvement in planning transition**		0.58		
**Parents’ satisfaction with resources**		0.46	0.37	
**Frequency of review of educational plan**			0.73	
**Parental involvement in defining transition goals**			0.65	
**Parental satisfaction with information given**		0.31	0.62	
**Extent organisations work together**			0.57	
**Extent plan developed ahead of transition**	0.34		0.43	
**Child’s involvement in decision making**				0.69
**Frequency of school visits prior to transition**				0.69
**Child’s involvement in transfer decision**				0.67
**Child’s involvement in defining transition goals**		-0.30	0.44	0.47
**Eigenvalues**	3.72	2.82	2.74	1.96
**% of Variance**	16.17	12.26	11.91	8.52

Principal component 1 (PC1), which we have summarized as ‘child inclusive ethos,’ includes questions such as child’s involvement in peer support and recreational activities, curriculum flexibility and staff’s ability to work as a team and the parents’ involvement in decision-making as well as the extent to which a transition plan was developed ahead of time and how well information was transferred from school to parents. PC1 contains 16.17% of the variation.

Principal component 2 (PC2), which represents child autonomy and involvement, is responsible for 8.52% of the total variation. It includes questions of child involvement in decision-making, frequency of school visits prior to transition, and the children’s involvement in the transfer decision and in defining transition goals. Additionally, PC2 includes some parental involvement in decision-making that was led by the child.

Principal component 3 (PC3) contains questions relating to parental involvement. Parents’ satisfaction with school resources and information provided by the school, their involvement in the transition, contact with school and other agencies, involvement in transfer decision as well as the accessibility of the school and the extent their child is adjusted are among the questions contributing positively to this component. The child’s involvement in defining transition goals contributed negatively to PC3. PC3 contributed to 12.26% of the overall variation.

Finally, principal component 4, which involves transition planning and coordination, contributed to 11.91% of the overall variation. Parents’ satisfaction with resources and information provided by the school, their involvement in defining transition goals, frequency of review of educational plan and the extent the transition plan was developed ahead of time formed this component.

Calculating a PCA demonstrates which variables correlate highly with one another by simplifying the structure underlying a large set of variables into a smaller set of components. A PCA does not show whether the components are related to any outcome. Thus, we calculated a logistic regression to see whether the components and the other variables in the data set were related to a successful transition.

In the logistic regression, all principal components were significantly related to a successful transition (PC1: OR 4.04, 95% CI 2.43–7.18; PC2: OR 2.15, 95% CI 1.34–3.57; PC3: OR 1.93, 95% CI 1.14–3.34, PC4: OR 1.88, 95% CI 1.24–2.98). Attendance at a special primary school was negatively associated with a successful transition (OR: 0.06, 95% CI 0.01–0.33, p<0.001). Attendance at other schools was not significantly associated with successful transition. Parents whose children were 6–8 years old were significantly associated with a positive transition (OR: 3.92, 95% CI 1.21–13.55, p = 0.026), though the confidence intervals in this estimate are quite wide. Language and gender were not significantly associated with the outcome and did not impact the model. The model was adjusted for country and additional support need. The results of the logistic regression can be found in **Table 4**.

**Table 4:** Relation of principal components and school attendance on successful transition.

Variable	Odds Ratio	95% CI	p-value
**Child Inclusive Ethos (PC1)**	4.04	2.43–7.18	<0.001
**Child Autonomy & Involvement (PC2)**	2.15	1.34–3.57	0.002
**Parental Involvement (PC3)**	1.93	1.14–3.34	0.016
**Planning & Coordination (PC4)**	1.88	1.23–2.98	0.005
**Mainstream Preschool**	Reference		
**Mainstream Primary School**	0.43	0.09–1.86	0.264
**Mainstream Secondary School**	0.39	0.07–1.97	0.262
**Special Class in a Mainstream Primary School**	0.94	0.17–5.11	0.943
**Special Class in a Mainstream Secondary School**	1.70	0.27–11.09	0.572
**Special Preschool**	0.34	0.06–1.95	0.229
**Special Primary School**	0.06	0.01–0.34	0.002
**Special Secondary School**	0.43	0.04–3.93	0.457
**Child age 3–5**	1.83	0.43–7.93	0.411
**Child age 6–8**	3.93	1.21–13.55	0.026
**Child age 9–11**	1.79	0.61–5.34	0.291
**Child age 12–13**	Reference		

## Discussion

Having an active child led ethos (PC1) by the parents and professionals in opening up their systems and processes does appear to be a main driver for successful transition of pupils with complex support needs into mainstream environments. PC1 contributed the most to a successful transition, though the confidence intervals were slightly wide (OR: 4.04; 95% CI: 2.43–7.18, p<0.001. The importance of having a process and systems that support a ‘child inclusive ethos,’ should not be underestimated. The three participatory process of child-led, parent- led and practitioner-led are evident within the PCA analysis. Children’s and adults’ rights are seen to complement each other, and as a result this can become one of the main factors for successful transition. Extrapolating this further, if one or the other becomes more dominant, such as an over-bearing parent, the impact of having a child ethos led transition could lessen the smoothness of the transition process itself and this interestingly, may be responsible the negative result in some primary schools (PC3).

The results confirm having a child inclusive ethos enables participation in decision making processes during transition. The key we believe to successfully supporting children with additional complex support needs is for all to be engaged in an active, proactive process. The process of inclusion requires that all parties engage in listening and making changes based on dialogue and when this is contextualised by a child led ethos, coupled with the barriers restricting the child centred engagement removed we see the power of the process to enable child/parent partnerships engage with practitioners to seek to balance individual, structural, power/political and cultural aspects of transition and inclusion.

Structural and cultural inclusion (PC1) rather than a focus on impairment appear to be important for successful transition. Flexible time-tables and curriculum that responded to children’s ideas rather than the other way round, allows for differences between children to emerge rather than a process which focuses on the normalisation of every child. Parents and children associated child led processes with flexible approaches to pedagogy. The parents promoted social inclusion and social interactions of children, with the need to balance specific/differentiated approaches with more generic community based pedagogy and the requirement for specific resources to be provided for the inclusion process.

The results of (PC1) in addition show being able to participate in wider school and community activities are enablers for greater successful transitioning from one environment to another. Participation in activities whether these are recreational, social and educational is the context in which children form friendships, develop skills and competencies, express creativity, and achieve mental and physical health [33–34]. We see in the transition process this is no different. Children with disabilities tend to be more restricted in their participation predominantly as it has been adult led, either by the professional or the parent or even both. However, if participation in the transition process of the child may be influenced by the child’s perceived self-competence [35], then more support should be given to the child in order to participate as an active engaged leader/participant of the transition process which could include such activities as buddy systems, children leading social activities and doing presentations on inclusion and joint visits with professionals where children could identify key issues to be resolved. Child-led transition can be the process by which children with additional complex support needs build upon their own confidence and independence and should not be seen as a process by which practitioners lead that we should differentiate between working processes that involve pupils and parents in genuine participation and practice that is sympathetic to concerns of all the actors involved in the process [36–39].

Though the effect is smaller than PC1, the results of PC2 demonstrate that sometimes children with complex support needs aspire to be treated the same as other children and at other times they wanted their diversity to be recognised (OR: 2.15: 95% CI: 1.34–3.57, p = 0.002). This means that professionals and parents need to spend time talking to children about the different contexts where they require different participatory engagement during the transition process.

Engaging in a shared experience is widely recognised as central to successful peer support particularly of parents of children with disabilities [40]. This, as our research suggests, is true for children with complex additional support needs. Involving children no matter the country to develop peer support systems as a factor to enhance their inclusion into mainstream environments is seen as critical. These peer collaborations can be composed of several categories of interaction such as peer awareness training, peer support arrangements, peer networks, peer tutoring [41]. Child-led transition includes buddy systems, children leading social activities and doing presentations on inclusion and joint visits with professionals where children could identify key issues to be resolved. One reason why peer support can facilitate better transition is that it can alleviate children’s fears and concerns and thus create a better, more familiar and relaxing environment which is one of the aims of creating successful transition.

Yet, in a previous report only 38.4% of professionals said that children were involved with defining the aims and outcome of the transition process and only 39.4% of professionals said that children were involved with the decision making processes of their own organisation [42]. Allowing children with complex additional support needs to be the driver of peer networks can also enhance the non-disabled peer of the relationships [43]. Enabling a process of child-led peer support systems within the structure of transition, that is to remove the barrier(s) that does not allow for this, is one way to strive for successful transitioning across European member states.

The research supports the idea that increased partnership (PC4) and planning before, during, and after process of transition enhances the likelihood of successful transition planning [44]. This was also significant, but the size of the actual effect was smaller (OR: 1.88; 95% CI: 1.23–2.98, p = 0.005). Thoughtful and detailed planning supported by good information and resources alongside frequent transition reviews with all involved with the transition process provides the settings that children and parents identified as being good on transition. Parents clearly value regular communication with professionals, however, what they value more is that this regular communication starts as early as possible in the transition process so that the sharing of information between networks, professional and parent child communities, can equally start as early. Supporting clear procedures, clarity of roles and clear vision and motivation (to include children) are important factors to sustain successful transition, in that bringing together participants in the transition process is central to the experience in order to provide opportunities to breakdown stereotypes, build mutual knowledge and understanding and consider how to work with differences throughout the process [45–47].

Table 5 provides a summary of answers to each of the five research questions based on the analysis of the survey questions.

**Table 5:** Summary answers to the research questions.

Research Question #	Question	Summary Answer
**1**	Is inclusion for all connected to structures, cultures and relationships that promote participatory discussion and collaborative problem solving between children, parents and staff [24–25]?	Schools that have a child inclusive ethos results (PC1, OR: 4.04, 95% CI: 2.43–7.18, p<0.001) in children participating in wider school and community activities; these are enablers for greater successful transitioning from one environment to anotherPC1 also demonstrates the importance of the three participatory processes of child-led, parent-led and practitioner-led process and support systems. Childrens’ and adults’ rights complement each other and as a result, this can become one of the main factors of transition
**2**	Does positive transition and inclusion for all result from children with complex additional support needs having autonomy and leading activities of transition within a context that promotes creative social relations [26]?	Positive transition and inclusion for all resulted in children having autonomy and led activities of transition. PC2 (OR: 2.15, 95% CI: 1.34–3.57, p = 0.002) shows how children with complex support needs aspire to be treated the same as other children, including leading on activities of transition. Giving children the autonomy to do this affords children different contexts where they can have participatory engagement during the transition process. Children’s involvement in decision-making, frequency of school visits prior to transition, and the children’s involvement in defining the transition goals were all contributors in a positive transition.
**3**	Is a child’s involvement in peer support and recreational activities key to positive transition and inclusion [27]?	PC2 also shows participating in wider school and community activities are enablers for greater successful transitioning from one environment to another. This is also supported by PC1 Having a child-led ethos within the school environment was reflected in peer support and recreational activities.
**4**	Does planning, provision of resources, development of flexible curriculum, teacher strategies and information sharing ahead of time lead to positive transition and inclusion [28]?	PC4 and PC1 in combination answered this question. Our analysis shows that structural and cultural inclusion, through planning and coordination, flexible timetables, increased partnership relations during the transition planning cycle rather than a focus on impairment appears to be important for successful transition.
**5**	Are participatory goal setting, early development of plans and regular review of plans and service delivery essential aspects of positive transition and inclusion [29–30]?	PC4 indicated that transition planning and coordination is an important component for successful transition. Parents’ satisfaction with resources and information provided by the school, the frequency of review of educational plan and the extent the transition plan was developed ahead of time provide support in ensuring positive transition

We believe the results of this paper has implications for some of the European Strategies that are currently being supported. For example, a key target for Europe 2020 is to reduce the level of early school leavers [48].] The EU Education and Training Monitors states “although comparative data is scarce, the available evidence states unambiguously that students hampered by a disability … are more likely to leave school before finishing upper secondary education” page 36 [49]. It is beyond the scope of this paper to claim that child led processes of transition would lessen the school leaving rates of children with disabilities, this is to be still empirically examined; however, ensuring that discussions about leaving school and entering into the next phase of the child’s life, are led by the child, and not adult focused, will at least allow for a holistic individual approach in developing a transition plan that considers the child and the family’s educational, psychological, cultural, social and daily living characteristics. These factors should become for the school as important as national and international performative regimes appear to be [50–51].

This work provides the evidence to enhance the recommendations made by Ebersold, Schmitt, Priestley on behalf of the Academic Network of European Disability Experts to European Governments [52]. They provide a series of nine recommendations to European governments of which four recommendations are empirically supported by this work; they argue that governments should

- Include transition issues in their education policies to ensure effective pathways from one educational level to another, from special schools to mainstream schools and from education to work.
- Provide the financing mechanisms necessary for effective and high quality education, transition opportunities and the support of innovative practices.
- Actively involve young disabled people, their parents and representative groups at all levels of educational policy making (both local and national)
- Ensure accessibility in a preventive manner including to teaching material and systems (page 13)

This work should encourage policy makers, both nationally (within partner countries) and internationally (EU2020) to implement fully the existing laws and conventions such as the UN Convention on the Rights of the Persons with Disabilities, (2006) Charter of Fundamental Rights of the EU, UNCRC 1989 It may be the case that no new laws need to be developed and passed within each country’s legislative system but simply implementing and monitoring fully the international agreements that governments have signed would be sufficient. The caveat to simply implementing international agreements though, is that research has shown that many countries have interpreted these international agreements in different ways [53–54] and particularly Davis and Deponio [55] have argued that there exists across countries a tension between implementing generic and specific laws to support the inclusion of children with disabilities. This work that has identified child and parent-led processes as a successful factor in the transition of children with disabilities, may resolve in part some of that tension by providing the bridge that joins specific approaches to inclusion with those that are based on more international and political solutions.

## Limitations

During the study design period, due to the use of translators and to employing individual surveys for each language, there were still some discrepencies between the languages. Although resulting from the pilot a uniformed approach was utilised, it became apparent during analysis that one of the translation services constructed the survey differently than the others (for example, some of the options for the answers were in a different order); however, we do not anticipate that this affected responses as this was corrected prior to analysis. In future, we will have surveys translated then back translated to ensure correct translation.

Unfortunately, we were unable to obtain equal representation from all countries surveyed despite our best efforts. This could be a result of selection bias, where parents from some countries were more willing to participate than others. In Spain, our survey was delivered in Catalan, rather than Spanish, and would not be generalizable across the entire country. Neither country nor language spoken were significant predictors of successful transition; however, it is possible that there were not enough cases within the less represented countries or languages to detect a difference. Future studies should make further efforts to include broader representation from parents from all countries. Despite these limitations, the study has still been able to successfully identify components that are associated with successful transition across settings, genders and ages of parents responding and children.

## Recommendations

We believe that from this research there are a number of recommendations that can be made both for children and parents, and school leaders and policy makers across Europe that have resulted from providing an answer to the 5 main research questions. It is important that the child who is at the centre of the transition processes ask questions about their new school, their new role within the school, and their new routines. In doing so, the child should be enabled (both independently and collaboratively) by parents and professionals to talk about their feelings regarding transition and this will include talking about their former school and decide with friends, teachers and parents on a suitable way to say good-bye.

Equally, it is just as important that the child makes clear their own priorities for their new school, examples could include stating what they like or dislike to do at school, their fears and their worries. Children should be made aware who their school contact person is in both their former and in their new school to support and listen to them as they go through the transition process. Children should be pro-active and talk about everything they want their teachers and peers to know about them beyond the child’s disability.

If the child is not able to follow the recommendations pro-actively and independently, it is the adults’–parents’ and professional’s role to find out about them and support the child in communicating them to the other stakeholders. None of these recommendations are country-dependent but are all driven by the four principal components of having a Child Inclusive Ethos (PC1) supported by having Child Autonomy and Involvement (PC2), followed by Parental Involvement (PC3), alongside Planning and Communication (PC4). Parents can initiate the development of a transition plan for their child and should be done early in advance so as to allow for all the decisions and adaptations to take place but should always put their children’s rights first in the process that is take into account their own child’s perspective. Parents need to be collaborative in the process, and need to know what their role (along with their child’s role) is in the transition process and that every other person involved is aware of their own role and related responsibilities in terms of time frame and expected results.

For school leaders and policy makers, we recommend that all professionals develop a transition strategy document for the school with clear procedures, time-lines, relevant agencies, target groups and indicators for success which should include procedures for pupils that are coming in-organization transition, for pupils leaving or out-of-school transitions, as well as transition within the school. Importantly, this strategy document must be developed in partnership with primarily the child, then the parents, and other relevant agencies.

From this research successful transition was afforded by applying a holistic individual approach in developing a transition plan for each child and that the professional considered the child and the family’s educational, psychological, cultural, social and daily living characteristics. By applying a community based approach the professional can ensure that the child will be included in their peer groups and develop processes that allow the family to keep in touch with other families. Schools need to raise the school’s staff capacity on transition and inclusion, by supporting staff training and information on:

○ Child led Transition management
○ State, local authorities and school’s procedures for assuring additional support
○ Team work with other professionals involved
○ Collaboration with parents
○ Along with information and training to support for the child with information, orientation, emotional support

Teachers from and to the child’s schools need to ensure that they participate actively in the transition plan development as a part of the transition team but importantly teachers need to make clear with the transition team their aspirations and concerns and seek for support from the child’s family, and other professionals. Teachers should ensure they are able to provide parents with up- to-date and clear information on transition and inclusion procedures and meet in-person with the family and the child early in advance to get used to each other and develop a relationship of trust and respect.

## Conclusion

In order to support a child with complex additional support requirements through transition from special school to mainstream (Nursery, Primary and or Secondary) the following should be adopted within each European Union Member State that all ensure children with additional support requirements and their parents are involved and are at the centre of all decisions that affect them. It is important that professionals recognise the educational, psychological, social and cultural contexts of a child with additional support requirements and their families which will provide a holistic approach to learning and remove barriers for learning. School leaders and policy makers should provide a transition framework which is flexible to the individual needs of children with additional support requirements and adaptable based on national policies needs to be developed which is tailor made and facilitate children with additional support requirements through bespoke approaches and pedagogy tailored to their individual needs whilst providing relevant, up to date and timely information to support children with additional and their parents in an accessible manner.

## Supporting information

S1 File Parent questionnaire. (DOCX)

S2 File Parent data. (CSV)
