# Supplementary material for: Factors associated with successful transition among children with disabilities in eight European countries
Source: PLoS One. 2017 Jun 21;12(6):e0179904. doi: 10.1371/journal.pone.0179904 (PMC5479584; doi:10.1371/journal.pone.0179904)
Supplement: S1 File — (DOCX) [file pone.0179904.s001.docx]

# Factors associated with successful transition among children with disabilities in eight European countries

# Parent Survey Questions

1. To what extent were you involved in planning your child’s transition to their current school?
   1. To a large extent
   2. To a certain extent
   3. Not sure
   4. To a limited extent
   5. Not at all
2. How often did you and your child visit the school prior to the beginning of the school year?
   1. Very often
   2. Often
   3. Neutral
   4. Rarely
   5. Very rarely
3. Which of the following professionals did you meet prior to your child transitioning? (can select more than one)
   1. Teacher
   2. Principal/Head Teacher
   3. Resource Teacher/Professional
   4. Special Needs Assistant
   5. Occupational Therapist
   6. Speech Language Therapist
   7. Social Worker
   8. Family Support Specialist
   9. Early Years Specialist
   10. Specialist Counsellor
   11. None of the Above
   12. Other
4. Was information about your child’s additional support needs transferred to their current school before transition took place?
   1. Yes
   2. No
   3. I don’t know
5. Were you involved in decided what school you child transitioned to?
   1. Very involved
   2. Involved
   3. Neutral
   4. Limited involvement
   5. Not involved at all
6. To what extent was your child involved in decided what school they transitioned to?
   1. Very involved
   2. Involved
   3. Neutral
   4. Limited involvement
   5. Not involved at all
7. Has a professional assessment ever been done to determine your child’s educational needs? (if no/NA – survey will jump to Q10)
   1. Yes
   2. No
   3. N/A
   4. No idea
8. Who completed this assessment? (can select more than one)
   1. Classroom teacher
   2. Support/specialist
   3. Social worker
   4. Local specialist
   5. Regional specialist
   6. National specialist
   7. Other (specify)
9. Following your child’s assessment, to what extent was a plan developed by professionals to deliver your child’s additional support needs?
   1. To a large extent
   2. To a certain extent
   3. Not sure
   4. To a limited extent
   5. Not at all
10. To what extent were you involved in the development of your child’s educational plan?
    1. To a large extent
    2. To a certain extent
    3. Not sure
    4. To a limited extent
    5. Not at all
11. How often is your child’s educational plan reviewed/evaluated?
    1. Very often
    2. Often
    3. Neutral
    4. Rarely
    5. Very rarely
12. To what extent was a specific transition plan developed for your child in advance of transition?
    1. To a large extent
    2. To a certain extent
    3. Not sure
    4. To a limited extent
    5. Not at all
13. What type of information was shared with you during the process of your child’s transition? (you can select more than one option)
    1. Admission’s policy
    2. Names and roles of school staff
    3. Curriculum information
    4. Support available
    5. Other (specify)
14. In what format was information given to you? (you can select more than one option)
    1. Information booklet
    2. Transition pack
    3. Leaflet
    4. Information sheet
    5. Other (please specify)
15. How satisfied were you with the information you received?
    1. Very satisfied
    2. Satisfied
    3. Neutral
    4. Unsatisfied
    5. Very unsatisfied
16. To what extent did you child receive child-friendly information?
    1. To a large extent
    2. To a certain extent
    3. Not sure
    4. To a limited extent
    5. Not at all
17. Did you attend meetings to talk through transition?
    1. Yes
    2. No
    3. NA
    4. There weren’t such meetings
18. Who participated in these meetings?
    1. Your child
    2. Special needs assistant
    3. Early years specialist
    4. Extended family
    5. Psychologist
    6. Family support specialist
    7. Class teacher
    8. Occupational therapist
    9. Specialist counselor
    10. Principal
    11. Speech and language therapist
    12. Resource teacher
    13. Social worker
    14. None of the above
    15. Other (specify)
19. How satisfied are you with the resources (time, teaching materials) provided for your child as a whole)?
    1. Very satisfied
    2. Satisfied
    3. Neutral
    4. Unsatisfied
    5. Very unsatisfied
20. To what extent do you think your child has adjusted to their new school?
    1. To a large extent
    2. To a certain extent
    3. Not sure
    4. To a limited extent
    5. Not at all
21. To what extent do you think your child’s staff work as a team
    1. To a large extent
    2. To a certain extent
    3. Not sure
    4. To a limited extent
    5. Not at all
22. How often do you contact the school professionals and other agencies that support your child’s needs?
    1. Very often
    2. Often
    3. Sometimes
    4. Rarely
    5. Very rarely
23. To what extent does your child’s school and other agencies work together to support your child’s needs?
    1. To a large extent
    2. To a certain extent
    3. Not sure
    4. To a limited extent
    5. Not at all (there is no agency involved, only the school)
24. Was your child’s transition coordinated by a key person in your child’s new school?
    1. Yes
    2. No
    3. NA
    4. No idea
25. To what extent were you involved in defining aims and outcomes with professionals during transition?
    1. To a large extent
    2. To a certain extent
    3. Not sure
    4. To a limited extent
    5. Not at all
26. To what extent was your child involved in defining aims and outcomes with professionals during transition?
    1. To a large extent
    2. To a certain extent
    3. Not sure
    4. To a limited extent
    5. Not at all
27. To what extent are you involved in decision making about your child in school?
    1. To a large extent
    2. To a certain extent
    3. Not sure
    4. To a limited extent
    5. Not at all
28. To what extent is your child involved in decisions that may affect them?
    1. To a large extent
    2. To a certain extent
    3. Not sure
    4. To a limited extent
    5. Not at all
29. How accessible do you consider the building/facilities of your child’s new school?
    1. Very accessible
    2. Accessible
    3. Neutral
    4. Inaccessible
    5. Very inaccessible
30. How inclusive do you consider your child’s new school
    1. Highly inclusive
    2. Relatively inclusive
    3. Neutral
    4. Limited inclusiveness
    5. Not inclusive
31. How flexible would you characterize your child’s current curriculum?
    1. Very flexible
    2. Flexible
    3. Neither
    4. Inflexible
    5. Very inflexible
32. To what extent do you think your child is included in social/recreational activities?
    1. To a large extent
    2. To a certain extent
    3. Not sure
    4. To a limited extent
    5. Not at all
33. To what extent is your child involved in (i.e. giving/receiving) peer support?
    1. To a large extent
    2. To a certain extent
    3. Not sure
    4. To a limited extent
    5. Not at all
34. Are you satisfied with your child’s transition process to their new school?
    1. To a large extent
    2. To a certain extent
    3. Not sure
    4. To a limited extent
    5. Not at all
35. What is your gender?
    1. Female
    2. Male
36. What country do you live in?
    1. Bulgaria
    2. Ireland
    3. Cyprus
    4. Romania
    5. Greece
    6. Spain
    7. Netherlands
    8. UK
37. Please specify the age of your child
    1. 3-5 years
    2. 6-8 years
    3. 9-11 years
    4. 12-13 years
38. Is your child male or female?
    1. Female
    2. Male
39. Does your child have one of the following additional support needs? (can select more than one)
    1. Physical additional support need
    2. Intellectual additional support need
    3. Sensory additional support need
    4. Learning additional support need
    5. Developmental delay
    6. Autism
    7. Unknown
    8. Other (please specify)
40. Which of the following school/classes is your child currently attending?
    1. Mainstream preschool
    2. Special preschool
    3. Mainstream primary school
    4. Special class in a mainstream primary school
    5. Special primary school
    6. Mainstream secondary school
    7. Special class in a mainstream secondary school
    8. Special secondary school
41. Is there anything else you would like to add? (text box)
